# Trauma-Focused Cognitive Behaviour Therapy in an Adolescent with Mixed-Dissociative Disorder: A Case Study

**DOI:** 10.1155/2023/1356682

**Published:** 2023-02-14

**Authors:** Ershad Hussain

**Affiliations:** Central Institute of Psychiatry, India

## Abstract

Dissociation is a psychopathological condition with a range of alterations or changes in the normally integrated mental functions such as identity, consciousness, or memory and can manifest in various types such as depersonalization, trance states, derealisation, dissociative amnesia, and dissociative identity disorder (Waller et al., 1996). From an etiological perspective, past experience of trauma plays a major role as a precipitating factor in dissociation, wherein dissociation is conceptualized as a reaction to trauma as a way to distance oneself from the traumatic situation and events related to it (Lanius et al., 2015). Initially understood as a coping mechanism, the persistent manifestation of dissociation hinders the overall psychological functioning and interferes with the daily activities of the individual. Therefore, the focus of the various treatment modalities of dissociative disorders is mostly focused on enhancing the coping skills of the patient. The current case study demonstrates the manifestation of dissociation in a 16 years old adolescent with a history of trauma. Furthermore, it documents the efficacy of Trauma-Focused Cognitive Statistical Manual of Mental (Cohen et al., 2017).

## 1. Background

Dissociation is a psychopathological condition with a range of alterations or changes in the normally integrated mental functions such as identity, consciousness, or memory and can manifest in various types such as depersonalization, trance states, derealisation, dissociative amnesia, and dissociative identity disorder [1]. The latest Diagnostic and Statistical Manual of Mental Disorders-fifth edition (DSM-5) describes dissociative disorders as broadly involving impairments in the integration of all of the following: consciousness, memory, identity, emotion, perception, body representation, motor control, and behaviour.

Dissociative disorders are rare in children, but conversion disorders account for the majority of them, especially in developing countries [2]. Studies have shown that dissociative disorders have a complex relationship with the patients' body, mind, and sociocultural environment [3], and those stressful life events, traumas, and adjustment difficulties have a positive association with subsequent dissociative and conversion symptoms in children.

From an etiological perspective, past experience of trauma plays a major role as a precipitating factor in dissociation, wherein dissociation is conceptualized as a reaction to trauma as a way to distance oneself from the traumatic situation and events related to it [4]. Initially understood as a coping mechanism, the persistent manifestation of dissociation hinders the overall psychological functioning and interferes with the daily activities of the individual. Therefore, the focus of the various treatment modalities of dissociative disorders is mostly focused on enhancing the coping skills of the patient. Trauma-Focused Cognitive Behavior Therapy (TF-CBT) is an evidence-based treatment approach shown to help children, adolescents, and their parents (or other caregivers) overcome trauma-related difficulties. It is designed to reduce negative emotional and behavioural responses following trauma, including child sexual abuse and other maltreatment, domestic violence, traumatic loss, mass disasters, multiple traumas, and other traumatic events.

The treatment addresses distorted or upsetting beliefs and attributions related to the traumas and provide a supportive environment in which children are encouraged to talk about their traumatic experiences and learn skills to help them cope with ordinary life stressors. The current case study demonstrates the manifestation of dissociation in a 16 years adolescent with a history of trauma. Furthermore, it documents the efficacy of Trauma-Focused Cognitive Behaviour Therapy (TF-CBT) in dissociation [5].

## 2. Case Report

Index patient, a 16 years old adolescent boy, belonging to Hindu nuclear family from a lower socioeconomic strata, hailing from rural Bihar was admitted in the inpatient Department of Central Institute of Psychiatry, Ranchi. The adolescent was maintaining well and would go to school regularly until about four months ago when, one day his cousin brother who was around one year older than him passed away by drowning in a river nearby his village, where he had gone to take bath. The adolescent was extremely shocked and traumatized when he came to know about this incident. He shared a close emotional bond with his cousin and would spend most of his time at school as well as home with his cousin brother. According to the adolescent, his deceased brother was like a father figure for him, who would always standby him in his good and bad times and would always save him from bullies in school.

The dead body of deceased brother was found two days later and the patient had a glimpse of the body, which left him completely shattered. The family members further observed a change in his behaviour; he would remain upset, stay alone, and would not talk to anybody in the following days.

The adolescent reportedly would have difficulty falling sleep and would stay awake and keep thinking about his deceased brother. When he would fall asleep, he would have nightmares causing him to wake up in a fearful state.

The adolescent resumed his schooling after a week and took the bus to his school. However, the family members received complaints about his behaviour as he would stay alone, not talk to anybody, and would not respond to his teachers either. Within few days, the adolescent experienced the first episode of dissociative amnesia, when one day he left his class without informing anyone and reached his aunt's place nearby his school. He would not speak to his aunt for hours. His parents were called and the patient was taken home. Later, upon inquiry, he could not recall anything about this journey to his aunt's place.

The adolescent experienced the second episode of dissociation, two days after this incident, the adolescent was studying inside his room, when his mother called him for some work, he was not present in the room and was not present around the home either. Hours after this, the family members received a call from a person who informed them about the adolescent. When the family members found him, he again reported being unable to recall the incident and further complained of dizziness and headache. He would also refuse to go to school and state that he cannot concentrate on his studies and that the image of deceased brother comes into his mind.

The adolescent had similar episodes 2-3 times in the week, whereby he would walk alone for distance of 1-2 kms and then suddenly ask people nearby about his own whereabouts. In one such incident, it was reported that the adolescent was walking with the mother to a market nearby. The mother noticed that after sometime, the adolescent walked by himself and did not respond to the mother and would not speak to anybody. His mother kept following him for 2-3 kms and suddenly the adolescent stopped and started asking the mother how he reached here or what are they doing here.

The family members were concerned about the deteriorating state of the patient. Eventually, they took him to a faith healer, who saw him and told them that he is being targeted by some evil spirit and asked them to perform rituals and did some religious spells. This further triggered a sense of fear in the adolescent, as he would say he is scared that something might happen to him.

In the following days, condition worsened as he would run away from home almost 3-4 times in a day without any recall. He also started having episodes where he would fall and lie down on the ground without any response and have jerky movements characterized by head shaking without any symptom of up rolling of eyes or tongue biting with his eyes tightly closed, that would last for a minute and occur every day. According to the mother, in one such incident, the adolescent had gone to take a bath by himself in the bathroom. After 3-4 minutes, the mother suddenly heard the adolescent screamed “mummy.” The mother tried to open the door but it was latched from inside. When, the adolescent's younger brother tried to peep in through the window, it was seen that the adolescent was lying still on the floor of the bathroom. They got inside and tried to wake him up, by calling him out loudly and by sprinkling water, but the adolescent did not respond in any manner and remained still. After 4-5 minutes, he got up and could recall anything about the incident. He again started asking the mother about the incident.

Eventually, family members brought him to outpatient department (OPD) of Central Institute of Psychiatry (CIP), and he was admitted eventually in the inpatient ward. His persistent and pervasive mood was irritable. His role functioning was impaired and personal care was intact.

### 2.1. Mental Status Examination

Index child was kempt, tidy, and in touch with surroundings. He was maintaining eye contact, rapport was established; speech was soft, relevant, coherent, and goal directed with normal reaction time and decreased productivity. He had adequate level of attention and concentration. Cognitive functions were intact. His affect was dysphoric, communicable, and appropriate.

Hopelessness, pessimistic view towards future was present in thought content, and no abnormality was detected in perception. His social judgment was impaired with grade II insight. The case was diagnosed as mixed-dissociative disorder as per the guidelines provided in the International Classification of Disease 10 (ICD 10).

### 2.2. Medical Investigation

The child had normal findings on Computed Tomography Scan (CT-Scan) and Electroencephalogram (EEG). Therefore, the possibility of seizures was ruled out.

### 2.3. Baseline Psychological Tests/Rating Scale Findings

#### 2.3.1. Wechsler's Intelligence Scale for Children-Iv

The Full-Scale IQ derived from the combination of all 10 subtest scores is considered to be the most representative estimate of global intellectual functioning. His Full-Scale IQ score is 89 which suggests a dull normal level of global intellectual functioning.

#### 2.3.2. The Severity of Dissociative Symptoms Scale

It also known as Brief Dissociative Experiences Scale (DES-B)—Modified is an 8-item measure that assesses the severity of dissociative experiences in children ages 11–17. Each item asks the child receiving care to rate the severity of his or her dissociative experiences during the past 7 days. Each item on the measure is rated on a 5-point scale (0 = not at all; 1 = once or twice; 2 = almost every day; 3 = about once a day, and 4 = more than once a day). The raw total score can range from 0 to 32, which is then converted into average total score by using the formula

(1) $$\begin{array}{c} \frac{\left(\text{Raw sum}\times 8\right)}{\text{Number of items that were actually answered}}. \end{array}$$

It allows the clinician to think of the severity of the child's brief dissociative experiences in terms of none (0), mild (1), moderate (2), severe (3), or extreme (4). The score and impression of the index child are as follows: The baseline raw score was 16 and the average score was 2, which indicates moderate level of dissociation symptoms.

### 2.4. Management Plan

(1) Target of therapy
  - To reduce anxiety and distress related to trauma
  - Increasing child's awareness of distorted appraisal of unwanted thoughts
  - To modify specific cognitive processes and behaviours that is maintaining the dissociative symptoms
  - To ward off negative emotions
  - To change dysfunctional parenting
(2) Treatment module: Trauma-Focused Cognitive Behaviour Therapy for Adolescents
(3) Techniques of Cognitive Behaviour Therapy
  - Psychoeducation (to build insight about the illness)
  - Relaxation (to reduce anxiety and distress related to trauma)
  - Thought-emotion-action matter (to modify his cognitive process and maintaining the dissociative symptoms)
  - Chill Out Kit (to ward off negative emotions)
  - Parent counseling (to change dysfunctional parenting behaviours)

#### 2.4.1. Rationale for Selected Mode of Therapy

Dissociation has been found to be linked with the experience of trauma [6]. Some authors have conceptualized dissociation as a complex response to the experience of trauma that is seen as adaptive in the context of trauma but understood as maladaptive in other settings [7, 8]. Therefore, treating dissociation includes acknowledging and treating underlying trauma response.

#### 2.4.2. Ethical Considerations

The informed consent was sought from the child as well as the parents regarding the adolescent's participation in the therapy as well as its documentation after the successful completion of nine sessions. Ethical guidelines of psychotherapy and research were followed during intervention as well as documentation of this case.

#### 2.4.3. Drug Prescriptions

The adolescent was prescribed with standardized dosage of antidepressant and antipsychotic, i.e., fluoxetine and olanzapine in the course of treatment.

#### 2.4.4. Intervention Module

The intervention was given by the author, who is an RCI Licensed Clinical Psychologist. The Trauma-Focused Cognitive Behaviour Therapy was delivered to the indexed patient in 9 sessions with duration of 1 hour each for a period of nine weeks in the inpatient ward. The initial session was mainly focused on building of therapeutic alliance with the adolescent and his mother who was the main caregiver during the adolescent's stay at the inpatient ward. In the first week, the clarification of history of presenting complaints was done with the adolescent's mother. The baseline assessment indicated that he has moderate level of distress and dissociative symptoms. The psychological causes of dissociation and role of TF-CBT in treatment was explained in details.

The second session was focused on teaching and practicing of Jacobson's progressive muscle relaxation training, which is one of the key techniques of TF-CBT commonly introduced in the initial sessions. The adolescent was also given homework to practice relaxation whenever he feels anxious and tensed in and around the inpatient ward. In the following week, the adolescent's attempts at relaxation practice were reviewed and he had practiced and reported a temporary relief in his body and mind with each round of JPMRT. The adolescent's attempts at relaxation were admired and he was further encouraged to practice it on a daily basis.

Once the rapport was adequately established and relaxation was properly taught to the adolescent, in the next session, his hobbies and likes were assessed. The adolescent was passionate about science projects and had received prize for his science project assignments at school, he liked socializing with other children, and drawing was also listed as one of his hobbies. An activity schedule was prepared for the adolescent collaboratively, which listed science project assignments, drawing, and interaction with other children in the inpatient ward along with the academic tasks. The adolescent was provide with the necessary materials for his drawing and science projects, including, sketch pens, drawing books, and science books from the ward stock. He was explained in details about the activity schedule and encouraged to follow it on a routine basis. In the fourth session, the concept of emotion, emotional identification, and emotional regulation was explained to the child with the help of emoticon stickers. He was taught to identify various emotions and rate his own emotions on the “emotional thermometer.” It was discovered that the major emotions experienced by the adolescent were anxiety and sadness, which were mostly related to his recent loss of his brother and uncertainty of his present condition.

In the next session, the concept of “Chill Out Kit” was explained to the adolescent. The therapist and the adolescent collaboratively included various activities including, drawing, talking to other children, practicing relaxation, and engaging in science projects into the child survival kit. He was taught about ways in which he can use these activities from the survival kit to ward off his anxiety or sadness. He was encouraged to use his survival kit as frequently as he can in the treatment ward. His attempts at using this kit were routinely monitored by the therapist in the ward. The sixth session focused on the thought aspect of TF-CBT, i.e., the Thought-Action-Matter. The meaning of thoughts and its relation with emotions and action were explained to the child with simple examples. He was explained if our thoughts are positive then our feelings and actions are also positive. But if our thoughts are negative, then our feelings and actions are also negative. The therapist and the adolescent worked on some activities based on Thought-Action-Matter aimed at helping the adolescent understand the thought-emotion link more clearly. The testing of thought for validity and utility was done in the next session collaboratively. The dominant negative thoughts including those related to “loss of his cousin” were put to test to assess the validity and utility of these thoughts. The adolescent was taught to restructure the thoughts by drawing from evidences “for and against” the thoughts. He was given homework based on the Thought-Action-Matter to deepen his understanding, to restructure his negative thoughts, and to develop a balanced, realistic, and adaptive thought.

In the following session, the mother was psychoeducated to develop a right attitude towards the adolescent by imparting correct information regarding nature, causes and management of dissociation with examples. An authoritarian parenting style was found from the mother's side. She was explained about the harmful effects of authoritarian parenting including shouting, critical comments, or hitting on the mental health of the adolescent. The mother was explained about the need to adopt an authoritative parenting style focused on placing reasonable demands on the adolescent by providing him with love and care without setting unrealistic expectations or punishment especially in academics.

In the ninth session, the activity on “survival kit” and “Thought-Action-Matter” was reviewed by the therapist and its importance was summarized to the adolescent. He was informed about the termination of the therapy and was given the homework to write about his own story of success, narrating about the various skills that he has learned in therapy. The adolescent maintained a record of his activities in survival kit and Thought-Action-Matter. It was observed that the adolescent was actively socializing and participating in the ward activities along with other children. The feedback was taken from the adolescent and the mother. Significant improvements in dissociative episodes were reported. The adolescent reported that he felt in control of his thoughts and that he was now aware of the activities and techniques that can be used to cope up and ward off distressing thoughts and emotions. The rating scale was readministered. The post intervention raw score was 2 and the average score was 0, which indicates no dissociative symptoms.

### 2.5. Discussion

In the present case, the dissociative symptoms were precipitated after the loss of adolescent's cousin brother, who was perceived as a father figure by him and was his primary support at home as well as school. The psychoanalytic theory postulates that the early loss of a parental figure can lead to psychopathological symptoms in children and also predispose them in adulthood to various psychiatric disorders [9]. Early loss of a close member can be traumatic and can overwhelm the coping responses of the child that can interfere with his self-regulation mechanisms [10]. Existing literature reports that children exposed to severe, chronic, and/or multiple-source trauma often present with dissociative symptoms which further impact their ability to do learn and socialize [7, 8, 11, 12]. Children who have little parental support due to parents who are absent, overwhelmed by their own posttraumatic stress, and dealing with psychiatric conditions are more vulnerable to dissociation, let alone if the caregiver is the cause of trauma [13]. In the present case, the adolescent presented with symptoms such as flashbacks, nightmares, and memories, typically seen in PTSD, but did not fulfill the criteria for a formal diagnosis of PTSD. Moreover, as per the existing literature, dissociative symptoms may sometimes also include posttraumatic symptoms such as avoidance and numbing, intrusive thoughts and memories, nightmares, flashbacks, traumatic reenactments, and hypnagogic hallucinations [14].

There are a number of treatment modalities available for trauma in adult population. However, these treatment options may not be as helpful for and adolescents as they have a lower awareness of emotions and struggle to express certain emotions. Given this, the Trauma-Focused Cognitive Behaviour Therapy for adolescents based on Cohen's model is simplified and applicable in adolescent population with a history of trauma. The intervention module includes parents or caregivers as well that plays a key role in management of psychopathology [15]. The existing literature in Trauma-Focused Cognitive Behaviour Therapy in and adolescents has mainly focused on its efficacy with posttraumatic stress disorder (PTSD) and posttraumatic stress syndrome (PTSS) wherein TF-CBT models are found to be more effective at decreasing PTSD and PTSS than waitlist control conditions, standard community care, child-centered therapy, and nondirective supportive therapy [16–18].

On the other hand, the existing studies on interventions in dissociative disorders in younger children includes play therapy, hypnosis, storytelling, and other forms of art therapy that does not require an advanced level of capacity to process and evaluate and restructure thoughts [19–21].

The existing literature on the efficacy of Trauma-Focused Cognitive Behaviour Therapy on dissociation is limited in both child as well as adolescent population. According to the author's knowledge, this is the first case study report that has demonstrated the clinical manifestation of mixed-dissociative disorder (with features of dissociative amnesia, dissociative convulsion, and occasional mood swings), and successful treatment adolescent. The short-term goals of the current intervention were to help to process painful memories of the loss, help him restructure his thoughts, and regulate his emotions. The long-term goal was to enhance the coping skills of the adolescent and prevent future episodes. The findings from the case study show that Trauma-Focused Cognitive Behaviour Therapy for adolescents as set out in the manual [5, 22] is effective in treating an adolescent with dissociation.

### 2.6. Limitations and Future Recommendations

Since it was a single case intervention report, the findings of the study may not be generalizable to a larger population with dissociative disorders. However, it facilitates the reader's conclusion about its transferability in cases with similar manifestations of dissociation. Secondly, as a part of the treatment as usual procedure (TAU), the child was already prescribed and adhered to psychotropic medications. Therefore, the clinical improvements cannot be fully attributed to the nonpharmacological intervention, i.e., (TF-CBT) delivered in the present case. Finally, the case report is based on a prepost intervention with no follow-up observation of the case. Therefore, the long-term efficacy of the TF-CBT in the present case could not be stated.

The future studies may focus on the efficacy of TF-CBT in adolescents in dissociative disorders with a larger sample size along with follow-up studies.
